# Comparative Evaluation of the Effectiveness of a Combination of Absorbable Gelatin Sponge and *Calendula officinalis* with Absorbable Gelatin Sponge Used Alone as a Hemostatic Agent—An *In-Vitro* Study

**DOI:** 10.3390/dj10050076

**Published:** 2022-05-05

**Authors:** Bharath Kumar Ayyanahalli Matta, Santhosh Kumar, Chetan Hasmukh Mehta, Usha Yogendra Nayak, Patricia Garcia Rodriguez

**Affiliations:** 1 Department of Periodontology, Manipal College of Dental Sciences, Manipal, Manipal Academy of Higher Education, Manipal 576104, India; dr.bharathkumar1995@gmail.com; 2 Department of Pharmaceutics, Manipal College of Pharmaceutical Sciences, Manipal Academy of Higher Education, Manipal 576104, India; chetan_jd12@rediffmail.com (C.H.M.); usha.nayak@manipal.edu (U.Y.N.); 3 Faculty of Medicine, Oncology, Vrije Universiteit, 1081 HV Amsterdam, The Netherlands; patricia.pgr@hotmail.com

**Keywords:** *Calendula officinalis*, cell proliferation, fourier-transform infrared spectroscopy, gelatin, hemorrhage, hemostatics

## Abstract

Excessive bleeding can complicate surgical intervention; this could be managed using an effective hemostatic agent that provides immediate and early bleeding control. Gelatin sponge and *Calendula officinalis* have been proven to have good hemostatic properties. The present *In-vitro* study analyzed the cytotoxicity and hemostatic properties of gelatin sponge and *Calendula officinalis*. The cytotoxic concentration/effective concentration of *Calendula officinalis* was determined by MTT (3-[4,5-dimethylthiazol-2-yl]-2,5 diphenyl tetrazolium bromide) assay. The drug release was determined using a vertical Franz diffusion cell apparatus; solid-state characterization was assessed using Fourier-transform infrared spectroscopy (FTIR) and a differential scanning calorimeter (DSC). The MTT assay showed 7% *Calendula officinalis* to be cytocompatible, and there was an increase in cell proliferation. When the 7% *Calendula officinalis* was loaded into the sponge, it was compatible, and the drug content was found to be 56.28 ± 13.84%. The time taken for the blood clot formation was measured using the Lee–White method. The gelatin sponge’s time for clot formation was 161.70 ± 3.11 s, and the *Calendula officinalis* loaded gelatin sponge’s time for clot formation was 158.75 ± 4.60 s. Hence, it could be concluded that when *Calendula officinalis* is incorporated into a gelatin sponge, it shows material compatibility and cytocompatibility, reduces the time for clot formation, and could be used as an alternative to other hemostatic agents.

## 1. Introduction

Excessive hemorrhage can be a significant complication both in healthy and medically compromised individuals, and it is also one of the most common complications during periodontal surgery [1]. It can be categorized as primary hemorrhage, reactionary hemorrhage, or secondary hemorrhage. Bleeding during surgery is known as a primary hemorrhage [2]. The bleeding that occurs two to three hours postoperatively is known as a reactionary hemorrhage, and if the bleeding occurs through fourteen days post-surgery, it is called a secondary hemorrhage.

Hemorrhages can vary based on injuries, such as an injury of the vasculature, injury of the bone, and soft tissue injury. In addition, the rate and severity of bleeding can be the consequence of a disease or a surgical procedure [3,4]. The bleeding complications can alter the outcome of periodontal plastic surgical procedures if not managed. Therefore, an effective hemostatic agent that provides immediate and early hemorrhage control is essential to avoid such adverse events during surgical procedures. The ideal topical hemostatic agent should be biologically acceptable, inexpensive, and efficient in managing bleeding [5]. Natural polymers such as gelatin sponge/foam have been used as a hemostatic agent for mild to moderate bleeding. Absorbable gelatin sponges are porous and are prepared from purified pork skin gelatin. They are available in many forms, such as films, square chips, absorbable sponges, and powder mixed with saline to form a paste [6]. Gelatin sponges are ideal as they are biologically inactive and are passive hemostats due to their three-dimensional structure [2]. They are non-toxic and are easily broken down into more minor compounds through a process called hydrolysis. They are liquefied in the oral cavity within a week and fully absorbed within four to six weeks. Absorbable gelatin biomaterials are easy to adapt and do not require effort, and they adhere to the wound’s surface, making them appropriate for application to irregular and uneven surfaces. They are generally used in emergency management since they are ready to use directly without clips, pins, or tacks; there is no need for a particular storage unit; and they are inexpensive [7]. Gelatin sponges can be used in dry and wet forms by moistening them with distilled water or normal saline [8,9].

In various developing countries, a large proportion of the population still depends on traditional herbal medicines to control bleeding; a few such medicines include *Punica granatum* L., *Myrtus communis* L., and Ankaferd [10,11]. *Calendula officinalis* belongs to the Asteraceae family and has a historical reference for its usage in folklore because of its rich heritage of ethnomedicinal properties. In addition, *Calendula officinalis* flowers have been known for their hemostatic properties [12]. It has also been reported that *Calendula officinalis* has enhanced blood-clotting properties due to the lutein and zeaxanthin present in its leaves [13]. Calendula extract has been used in mouthwash and toothpaste to control gingival bleeding, and it has wound-healing, anti-inflammation, soothing, and unique cell-rejuvenation properties [14,15]. There are many studies in which *Calendula officinalis* has been used in different concentrations as a hemostatic agent [16,17,18]. Since the gelatin sponge is porous, the ultrastructural mesh-like framework of the material helps to expand and hold the clot and assists in coagulation. According to our hypothesis, the incorporation of *Calendula officinalis* into a gelatin sponge could aid in faster clot formation. Hence, our study aimed to identify the cytotoxic concentration of *Calendula officinalis* oil using the MTT assay, incorporate the non-toxic or effective *Calendula officinalis* into a gelatin sponge, and test its effectiveness as a hemostatic agent. The first objective was to evaluate the cytotoxicity of the *Calendula officinalis* oil on human gingival fibroblast cells (hGf) using the MTT assay. The second objective was to develop and characterize the *Calendula officinalis* loaded gelatin sponge and assess the blood-clotting property.

## 2. Materials and Methods

### 2.1. Materials

*Calendula officinalis* oil was purchased from AOS Products Private Ltd., Ghaziabad, India. A CuraSpon CS-950 sponge was supplied by CuraMedical B.V., Assendelft, The Netherlands. Ethanol and potassium dihydrogen orthophosphate were purchased from Finar Limited, Ahmedabad, India. Sodium hydroxide pellets were obtained from HiMedia Laboratories Pvt. Ltd., Mumbai, India. Gingival tissue from patients undergoing gingivectomy was used with prior consent to obtain human gingival fibroblast cell lines (hGf). The tissues were anonymized before they were sent for cell culture. These were maintained at the Manipal School of Life Sciences, Manipal Academy of Higher Education, Manipal, India. Dulbecco’s Modified Eagle Medium (DMEM), Leibovitz’s L15 Medium, fetal bovine serum (FBS), and trypsin were obtained from HiMedia Laboratories Pvt. Ltd., Bombay, India. Dimethyl sulfoxide, EDTA, and ethanol were purchased from Sigma-Aldrich Corporation, St. Louis, MO, USA. All the reagents that were used in the study were of analytical grade.

### 2.2. Method

#### 2.2.1. *In-Vitro* Cytotoxicity Assay

According to the standard cell culture procedures, hGf cells were cultured and maintained at the Manipal School of Life Sciences, Manipal Academy of Higher Education, Manipal, India. The fibroblast cells were cultured in the Dulbecco’s Modified Eagle Medium containing 10% FBS, and the cultures were incubated at 37 °C in a humid atmosphere of 5% CO_2_ in an incubator. The cells were then passaged and frozen until treatment. The cytotoxicity of the *Calendula officinalis* oil was tested *In-vitro* on the hGf cells using MTT (3-(4,5-dimethylthiazol-2-yl)-2,5-diphenyltetrazolium bromide) tetrazolium reduction assay [19]. About 1 × 10^4^ cells/well in 100 µL media were seeded into a 96-well culture plate and allowed to attach to the plate for 24 h. The cells were treated with 2%, 5%, 7%, or 10% concentrations of *Calendula officinalis* oil. The stock solution was prepared such that the concentration of DMSO in *Calendula officinalis* did not exceed 0.1%. (This concentration of DMSO did not affect cell viability.) Human gingival fibroblast cell lines incubated in a culture medium without an active agent served as a control. The test was done in triplicates on the same batch of cells. The cell survival percentage was then calculated using the following formula:

$$\mathrm{Percentage}\;(\%)\;\mathrm{of}\;\mathrm{cell}\;\mathrm{survival}=\frac{\mathrm{Mean}\;\mathrm{abs}\;\mathrm{of}\;\mathrm{triplicates}\;\mathrm{drug}\;\mathrm{wells}\;}{\mathrm{Mean}\;\mathrm{abs}\;\mathrm{of}\;\mathrm{triplicates}\;\mathrm{control}\;\mathrm{wells}}\;\times \;100$$

**Figure 1:**
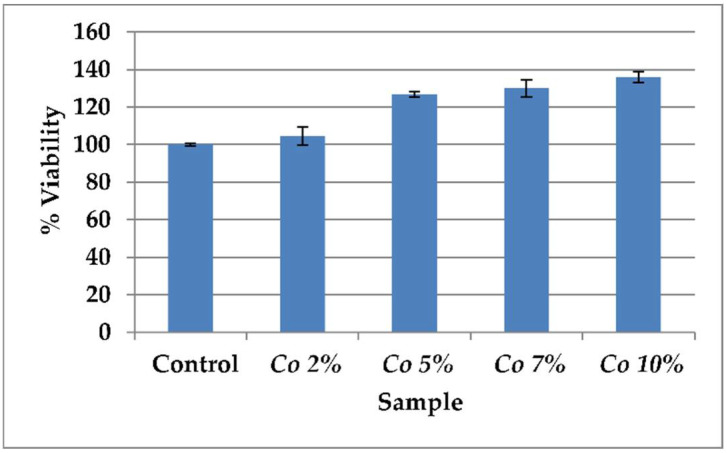
Percentage of cell viability at various concentrations of *Calendula officinalis* oil.

A bar chart was plotted using the percentage of the cells that survived against the concentration of the *Calendula officinalis*, and the IC50 values of aliquots extracts were calculated [20] (Figure 1).

#### 2.2.2. Preparation and Evaluation of *Calendula officinalis* Oil-Loaded Gelatin Sponges

Two different methods were followed to prepare oil-loaded sponges. The first method was to weigh the sponge using a sensitive weighing balance and add the required quantity of the *Calendula officinalis* oil homogeneously to the sponge (2 cm × 2 cm). The oil-loaded sponge was transferred to the labeled Petri dishes and dried in the oven at 45 °C. The sponges were weighed at different time intervals (6 h, 12 h, 24 h, 36 h, 48 h, and 72 h) to check changes during drying, such as weight loss and morphological changes. The second method was too weigh the sponge and the required amount of oil in Eppendorf tube using a sensitive weighing balance followed by dispersion of oil into 100 µL of ethanol.. The ethanolic mixture of oil was added to the pre-weighed sponge homogeneously and kept for drying at room temperature (30–37 °C). Again, the sponge was weighed at different time intervals of 6 h, 12 h, 24 h, 36 h, 48 h, and 72 h [21,22].

#### 2.2.3. Drug Content

The *Calendula officinalis* loaded sponge was dipped into the ethanol and sonicated for one hour to extract the oil from the sponges. The samples were analyzed for the presence of the *Calendula officinalis* oil in the sponges using a UV spectrophotometer at 237 nm, and the concentration was calculated using a prepared calibration curve plot [23].

#### 2.2.4. Solid-State Characterization of *Calendula officinalis* Oil-Loaded Sponge

##### Fourier-Transform Infrared Spectroscopy (FTIR)

Fourier-transform infrared spectroscopy (FTIR) spectra were recorded in the 4000 to 500 cm^−1^ range using a Bruker ALPHA II FT-IR spectrophotometer. During this procedure, the sample was kept entirely on the ATR crystal, and slight pressure was applied with the pressure rod to bring the crystals into contact with the sample. The crystal was used to transmit the infrared light. The light then interacted with the sample, and the detector collected the reflected light, which was then used to generate the spectrum [24].

##### Differential Scanning Calorimetry (DSC)

A Shimadzu DSC-60 was used for the DSC analysis of pure *Calendula officinalis* oil, gel sponges, and gel sponges loaded with *Calendula officinalis*. A weighed sample was placed in a sealed aluminum pan and heated from 25 °C to 350 °C at a scanning rate of 5 °C per min under nitrogen flow (30 mL/min). An empty aluminum pan was used as a benchmark. Pure *Calendula officinalis* oil, a gelatin sponge, and a *Calendula officinalis* loaded gelatin sponge had their heat flow measured as a function of temperature [25].

##### *In-Vitro* Diffusion Study

The *In-vitro* drug diffusion study was carried out using a vertical Franz diffusion cell apparatus. The receptor compartment was kept in contact with the semipermeable membrane by being filled with simulated saliva solution (pH 6.75) (dialysis membrane, HiMedia Laboratories Pvt. Ltd., Mumbai, India, 12 KDa). The semipermeable membrane was firmly encased between the donor and receptor compartments. A sponge (2 cm × 2 cm, 7 percent loaded) and pure *Calendula officinalis* oil (equivalent to 7 percent loaded into the sponge) were placed on the semipermeable membrane. The speed of rotation used throughout the procedure was 400 revolutions per minute, which included periodic removal of 1-milliliter samples (0.25, 0.5, 0.75, 1, 2, 3, 4, 6, 8, 12, 24, 36, 48, 60, and 72 h). The diffusion profile was plotted after the samples were analyzed with UV spectroph.otometry at 237 nm [26].

#### 2.2.5. Hemostasis *In-Vitro* (Clotting Time Determination)

Approximately two milliliters of fresh human blood were added to each of the four polypropylene tubes containing the *Calendula officinalis* oil, *Calendula officinalis* oil-loaded sponge, gelatin sponge, and control. Each group was tested in triplicate and compared, with the fresh blood used as a control. We started with the null hypothesis that there was no significant difference between the groups.

The coagulation time was started when the four tubes were rapidly vibrated for a few seconds. The solution in each tube was then mixed with 300 microliters of calcium chloride. All test tubes were incubated at 37 °C and titled 45° for every couple of seconds; the clotting time was measured when a healthy clot was detected [27,28,29].

## 3. Results

### 3.1. Cytotoxicity Assay

Cell survival in the negative control was near 100%, and in the presence of *Calendula officinalis* oil, cell viability was 99.4%. The cell viability assay suggested that *Calendula officinalis* generally has low cytotoxicity to hGf cells. Further, as the concentration increased above 7%, no significant difference was observed in the cell viability, but the media showed precipitation of the oil that interfered with the proliferation of hGf cells. Hence, 7% was considered the optimal concentration for further analysis (Table 1).

### 3.2. Preparation of Calendula Oil-Loaded Sponge

**Figure 2:**
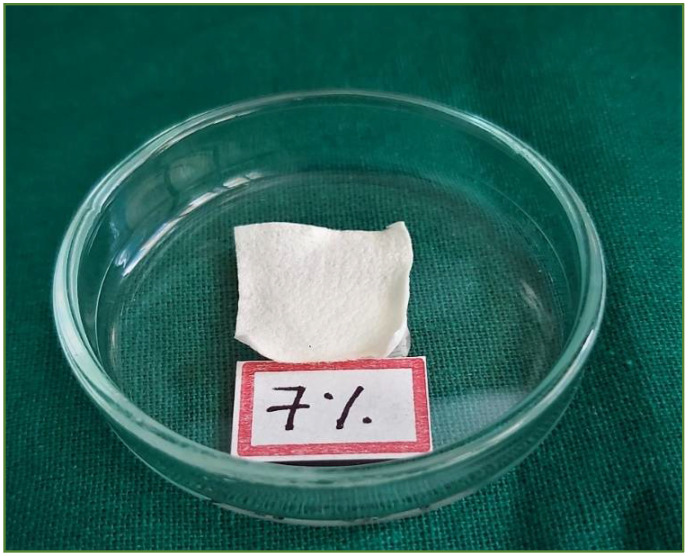
*Calendula officinalis* loaded gelatin sponge.

Based on the results, we concluded that the second method was better as it helped to prepare the oil-loaded sponges successfully without affecting their morphological structure. Therefore, 7% oil-loaded sponges were considered for further evaluation and characterization. Furthermore, based on the drying time study, we observed that the oil-loaded sponge using the second method dried in almost six hours, which was considered final for further preparation and drying of the sponge (Figure 2).

### 3.3. Drug Content

The UV spectrophotometric analysis showed the *Calendula officinalis* content of 56.28 ± 13.84% in the gelatin sponge.

### 3.4. Evaluation and Characterization of Calendula Oil-Loaded Sponge

#### 3.4.1. Fourier-Transform Infrared Spectroscopy (FTIR)

**Figure 3:**
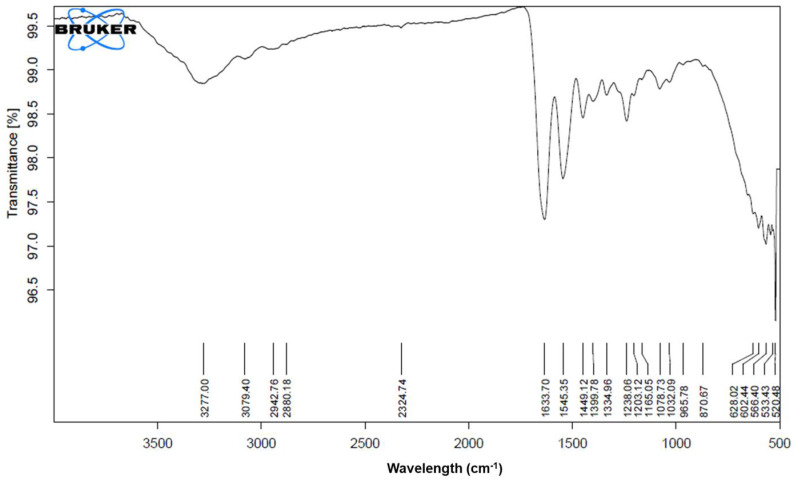
FTIR of gelatin sponge.

**Figure 4:**
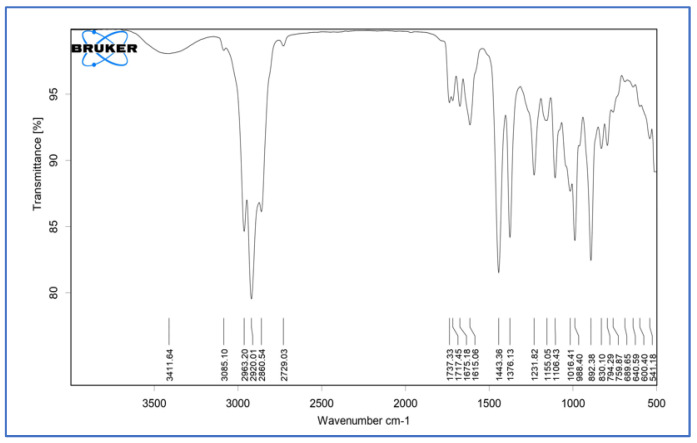
FTIR of *Calendula officinalis* oil.

**Figure 5:**
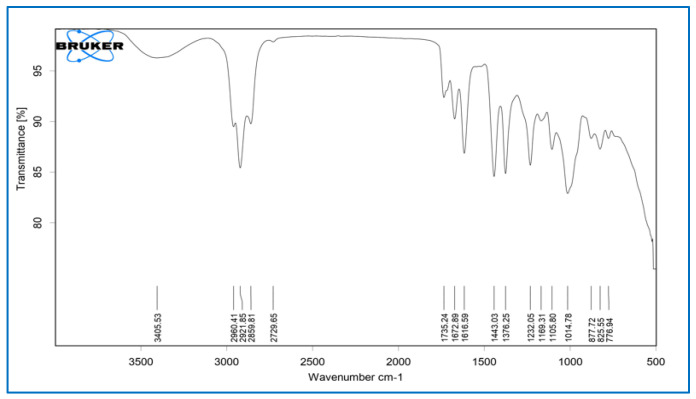
FTIR of *Calendula officinalis* oil-loaded gelatin sponge.

The pure *Calendula officinalis* oil showed characteristic peaks at 3411.64 cm^−1^, 2920.01 cm^−1^, 2860.54 cm^−1^, 1737.33 cm^−1^, 1615.06 cm^−1^, 1443.36 cm^−1^, 1231.82 cm^−1^, 1106.43 cm^−1^, 640.59 cm^−1^, and 541.18 cm^−1^, which represented the presence of alcohols and phenols (O-H alkanes, H-bonded), carboxylic acid (O-H), alkanes (C-H stretch), ketones and saturated aliphatic (C=O stretch), amines (N-H stretch), alkanes (C-H bend), alkyl halides (C-H wag), aliphatic amine (C-N stretch), and alkyl halide (C-Br stretch). The *Calendula officinalis* loaded gelatin sponge showed the retention of pure *Calendula officinalis* peaks in the sponge formulation at 3405.53 cm^−1^, 2921.85 cm^−1^, 2859.81 cm^−1^, 1735.24 cm^−1^, 1616.59 cm^−1^, 1443.03 cm^−1^, 1232.05 cm^−1^, and 1105.80 cm^−1^. The retention of characteristic peaks of *Calendula officinalis* in formulated sponges proved no incompatibility between the oil and sponges after formulation (Figure 3, Figure 4 and Figure 5).

#### 3.4.2. Differential Scanning Calorimetry (DSC)

**Figure 6:**
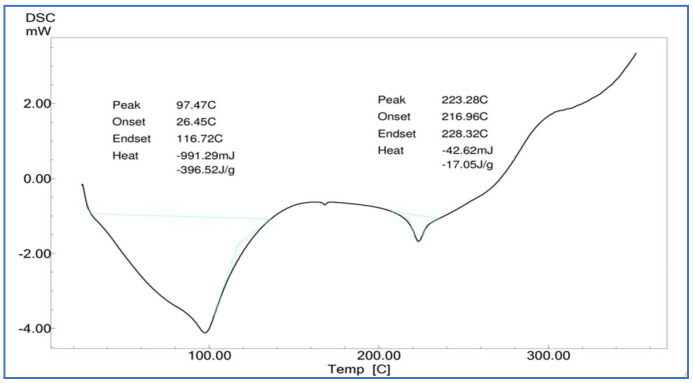
DSC of gelatin sponge.

**Figure 7:**
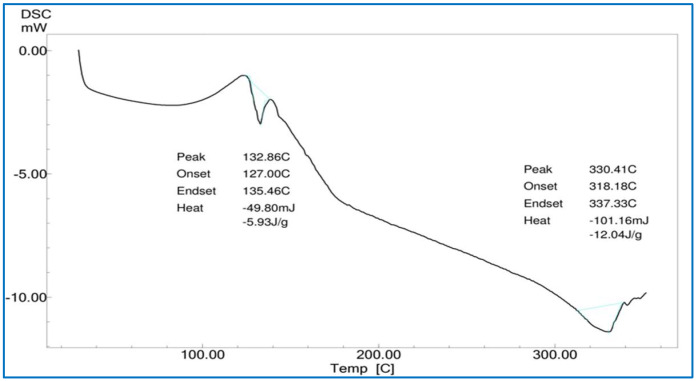
DSC of *Calendula officinalis* oil.

**Figure 8:**
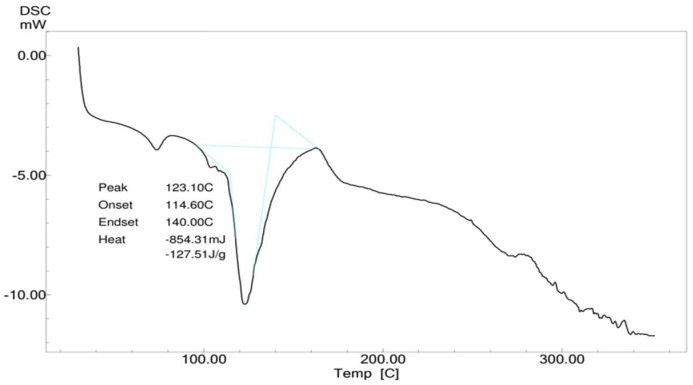
DSC of *Calendula officinalis* oil-loaded gelatin sponge.

The pure Calendula oil showed endothermic peaks at 132.86 °C and 330.41 °C, and the *Calendula officinalis* loaded gelatin sponge showed a sharp endothermic peak at 123.10 °C, which indicated slight shifting of the endothermic peak, perhaps due to the interaction of *Calendula officinalis* with the gelatin sponge (Figure 6, Figure 7 and Figure 8).

#### 3.4.3. *In-Vitro* Diffusion study

**Figure 9:**
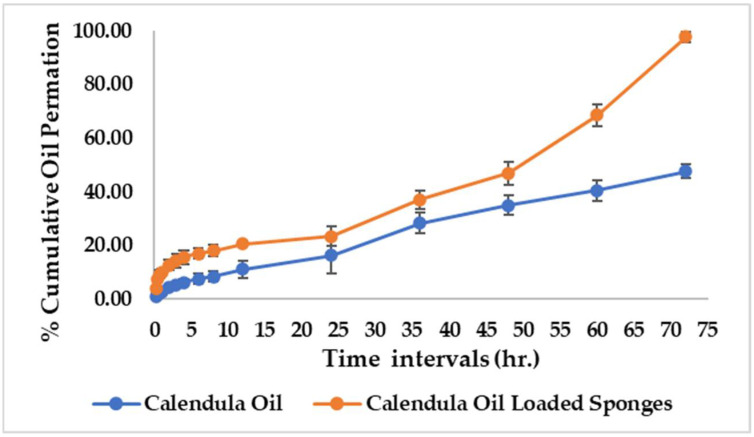
*In-vitro* diffusion profile of pure *Calendula officinalis* oil and *Calendula officinalis* oil-loaded sponge.

The diffusion profile showed a faster release of oil from the sponge when compared with pure oil, which indicated that the prepared sponge loaded with the oil was the best formulation technique to provide a therapeutic effect in treatment and enabled the oil to sustain release for a longer time. The *In-vitro* diffusion profiles of pure *Calendula officinalis* oil and the *Calendula officinalis* oil-loaded sponge are shown in Figure 9.

### 3.5. Hemostasis In-Vitro (Clotting Time Determination)

**Figure 10:**
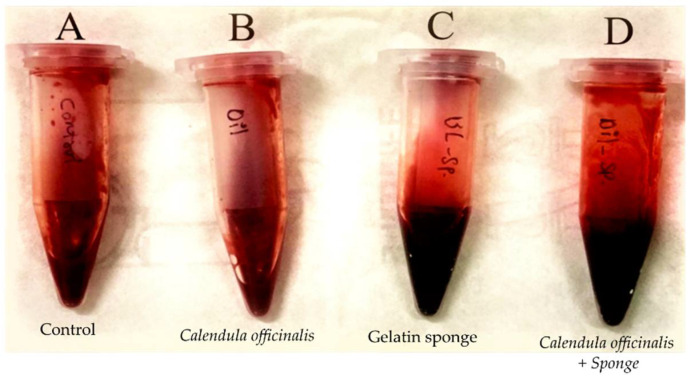
Blood clot formation using Lee–White method. Control tube (**A**) showing Blood clot formed that does not contain any agents, tube (**B**) with blood clot containing *Calendula officinalis* oil, tube (**C**) contains gelatin sponge, and tube (**D**) containing the test agent 7% *Calendula officinalis* oil incorporated in gelatin sponge.

**Figure 11:**
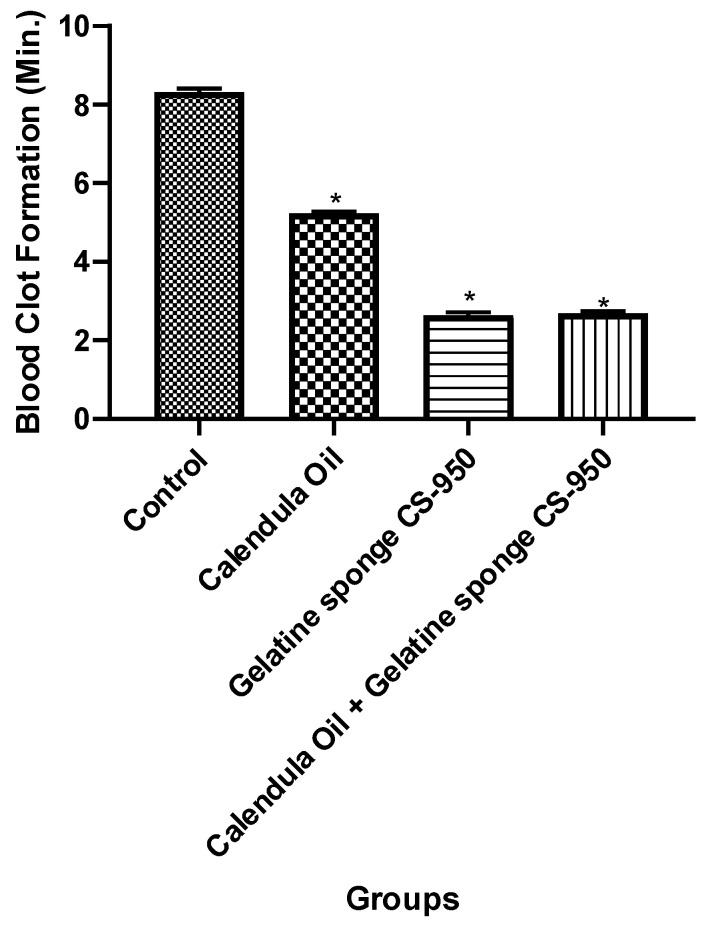
The bar graph result of comparison between the duration required for the blood clot formation within the 4 groups, *—showing statistically significant difference (*p* < 0.001).

The results showed that there was a significant difference between the groups (Figure 10 and Figure 11). Hence, the null hypothesis was rejected, and the alternate hypothesis was accepted. The time taken for the formation of a clot in the control group was 499.7 ± 5.37 (8.32 ± 0.089 min); in the presence of a gelatin sponge, the time decreased to 158.75 ± 4.60 s (2.64 ± 0.076 min), whereas the time in the presence of the *Calendula officinalis* loaded gelatin sponge was 161.70 ± 3.11 s (2.69 ± 0.051 min). Hence, it could be inferred that there was a considerable difference between the clotting time in the presence of the gelatin sponge or *Calendula officinalis* loaded gelatin sponge. However, the difference between the *Calendula officinalis* loaded gelatin sponge and the gelatin sponge alone was negligible. (Figure 11 and Table 2).

## 4. Discussion

Pharmacological investigations have shown that the *Calendula officinalis* extract possesses clotting properties, and it is also known to decrease the prothrombin time during the formation of a clot [13,18]. Furthermore, several clinical trials using *Calendula officinalis* have shown it to be an effective oral bleeding control agent during surgical wound management [14,30,31]. The results of our study demonstrate that *Calendula officinalis* oil is biocompatible. In addition, there was no cytotoxic effect on hGf t cells, and the proliferation of cells increased as the oil concentration was increased. The results follow the study done by Preethi, et al. [32], and Lagarto, et al. [33]. In our study, we considered 2%, 5%, 7%, and 10% concentrations of the *Calendula officinalis* oil based on evidence from the studies on animals and hGf cells by Shafeie, et al., and Saini P., et al., who had concluded that 7% *Calendula officinalis* was better than the 2%, 5%, and 10% concentrations. Similarly, the results of our study showed 7% of *Calendula officinalis* oil to have more significant proliferation of fibroblasts compared with 2% and 5% [34,35].

*Calendula officinalis* has flavonoids, many alkaloids, and triterpenoids as phytochemical constituents. It has shown proliferation of HSF (human skin fibroblasts) and has shown to have anti-inflammatory, antioxidant activity in a comparative study [36]. Our study combined *Calendula officinalis* with a gelatin sponge; this medicinal plant has also been combined with chitosan and silver nanoparticles as a wound-healing agent [37]. It has been shown to have good healing capacity for vascular injuries. However, in another study on open palatal wounds, the application of a *Calendula officinalis* based topical agent showed healing similar to that of the oxidized regenerated cellulose. In this study, only a topical agent of *Calendula officinalis* was used, which could have had a momentary effect [38].

A study was conducted by Menda JP, et al., to characterize and prepare a wound dressing agent by incorporating chitosan with *Calendula officinalis* extract, and it was seen that amino peak shifted from 1638 cm^−1^ to 1640 cm^−1^. In addition, the hydroxyl peak shifted from 3249 cm^−1^ to 3209 cm^−1^, similarly to our study with a peak hydroxyl shift from 3411.64 cm^−1^ to 3405.53 cm^−1^ and an amino peak shift from 1443.36 cm^−1^ to 1443.03 cm^−1^. Hence, we could infer that when the *Calendula officinalis* oil was incorporated into the gelatin sponge, there was a complete absorbance of oil into the sponge, and it was linear and evenly distributed [22]. Furthermore, the DSC value of the *Calendula officinalis* oil and *Calendula officinalis* loaded gelatin sponge showed endothermic peaks at 132.86 °C and 123.10 °C, respectively, suggesting good compatibility between the two materials. Similar results were seen in a study conducted by Lide Arana, et al.; the DSC values showed a clear endothermic signal corresponding to the solid and fluid transitions, with a midpoint transition of temperatures between 60 and 72 °C [26].

Our study demonstrated the hemostatic property using the Lee–White method for clotting time [39]. The time difference between the formation of the clot in the presence of the gelatin sponge and the *Calendula officinalis* loaded gelatin sponge was 2.97 ± 2.78 s. The difference in the time was a couple of seconds, and incorporation of the oil showed little difference as a hemostatic agent during intraoperative control of bleeding. The difference in the duration of clot formation compared with the control was 340.95 ± 3.16 s (~5 min 40 sec) and 338 ± 2.91 (~5 min 38 sec) when the *Calendula officinalis* was used in combination with the sponge and when gelatin sponge was used alone, respectively. *Calendula officinalis* belongs to an Asteraceae group of medicinal plants that can decrease the clotting time and bleeding time [40,41]. Hence, using such hemostatic agents can play a crucial role in the emergency control of hemorrhage following various types of tissue injury and decreasing the associated mortality and morbidity [42,43].

Gelatin sponges are soft and flexible biomaterials with a well-ordered micropore structure. This one-of-a-kind structural property demonstrates superior fluid permeability, cell interaction, and interfacial properties. However, they are mechanically fragile and cannot retain the desired and designated shape until new tissue is formed. The pores in gelatin allow it to be an excellent carrier of *Calendula officinalis* oil [44], and the material conforms easily to wounds and irregular surfaces. When it comes to coagulation cascades, gelatin-based biomaterials act physically rather than chemically in the gelatin sponge. The fibroblasts in cell cultures have shown no toxic effects to gelatin sponges. It is an excellent topical hemostat due to its low cost, ease of use, and good hemostatic activity. The gelatin matrix has been used to reduce post-surgical morbidity following dental extractions and periodontal surgeries and has been widely accepted [42,43]. In order to boost cell proliferation and hemostasis, we used *Calendula officinalis* oil infused into a gelatin sponge. Periodontal surgeries, emergency rooms, and war zones could benefit from gelatin sponges infused with *Calendula officinalis* [29,45,46,47,48].

A few of the limitations of our study include failure to analyze the type/characteristics of clot formed in *Calendula officinalis* incorporated gelatin sponge group. Second, we employed the whole extract of *Calendula officinalis* without any separation of natural chemical structures. Further, the result must be interpreted with caution as it was an *In-vitro* study. Future work in this direction includes analysis of the mechanical property of the combination, use of different forms of *Calendula officinalis,* and analysis of a higher concentration of the *Calendula officinalis* oil since we had seen an increased proliferation of fibroblasts as the concentration increased. Finally, a shelf-life analysis of the *Calendula officinalis* incorporated sponge would have given us a better understanding of the biomaterial. Though there was not much difference between the clotting times of the gelatin sponge and the *Calendula officinalis* oil-incorporated gelatin sponge, the oil-incorporated gelatin sponge’s advantages in terms of healing and other clinical parameters need to be determined in clinical trials.

## 5. Conclusions

In this study, the cytotoxicity assay of *Calendula officinalis* showed 7% concentration to be optimum for cell proliferation and for incorporation into the gelatin sponge. This combination was compatible as observed in FTIR and DSC. There was an even distribution of *Calendula officinalis* in the gelatin sponge as determined by the drug content. In addition, the combination showed an early clot formation. Thus, the gelatin sponge incorporated with 7% *Calendula officinalis* showed cell proliferation and was a hemostatic agent.

## Figures and Tables

**Table 1:** Human gingival fibroblast cells’ viability by MTT assay.

Sample	% Viability
Control	100 ± 0.5
2% *Calendula officinalis*	104.7 ± 4.9
5% *Calendula officinalis*	126.7 ± 1.5
7% *Calendula officinalis*	130.1 ± 4.5
10% *Calendula officinalis*	136.0 ± 3.0

**Table 2:** Clotting time assessed using Lee–White method.

Groups	Clotting Time (Seconds)	Clotting Time (Minutes)
Control	499.7 ± 5.37	8.32 ± 0.089
*Calendula officinalis* oil + gelatin sponge	161.70 ± 3.11	2.69 ± 0.051
Gelatin sponge	158.75 ± 4.60	2.64 ± 0.076
*Calendula officinalis* oil	314.30 ± 2.40	5.23 ± 0.04

## Data Availability

Not applicable.

## References

[1] Paepe A.D., Malfait F. (2004). Bleeding and bruising in patients with Ehlers-Danlos syndrome and other collagen vascular disorders. Br. J. Haematol..

[2] Mani A., Anarthe R., Kale P., Maniyar S., Anuraga S. (2018). Hemostatic agents in dentistry. Galore Int. J. Health Sci. Res..

[3] Werner E.J., Broxson E.H., Tucker E.L., Giroux D.S., Shults J., Abshire T.C. (1993). Prevalence of von Willebrand disease in children: A multiethnic study. J. Pediatr..

[4] Kumar M., Pai K.M., Vineetha R., Kurien A. (2020). Oral hygiene and dentition status in patients with congenital hemorrhagic disorders: A comparative study. Bras. Odontopediatria Clínica Integr..

[5] Brodbelt A.R., Miles J.B., Foy P.M., Broome J.C. (2002). Intraspinal oxidised cellulose (Surgicel) causing delayed paraplegia after thoracotomy—A report of three cases. Ann. R. Coll. Surg. Engl..

[6] Hiwatashi N., Hirano S., Mizuta M., Tateya I., Kanemaru S., Nakamura T., Ito J., Kawai K., Suzuki S. (2015). Biocompatibility and efficacy of collagen/gelatin sponge scaffold with sustained release of basic fibroblast growth factor on vocal fold fibroblasts in 3-dimensional culture. Ann. Otol. Rhinol. Laryngol..

[7] Spotnitz W.D., Burks S. (2008). Hemostats, sealants, and adhesives: Components of the surgical toolbox. Transfusion.

[8] Rodrigues R.N., Lopes A.A., Torres J.M., Mundim M.F., Silva L.L., Silva B.R. (2015). Compressive neuropathy of the first branch of the lateral plantar nerve: A study by magnetic resonance imaging. Radiol. Bras..

[9] Szpalski M., Gunzburg R., Sztern B. (2004). An overview of blood-sparing techniques used in spine surgery during the perioperative period. Eur. Spine J..

[10] Javan R., Yousefi M., Nazari S.M., Amiri P., Mosavi-Jarrahi A., Modiramani P., Naghedi-Baghdar H. (2016). Herbal Medicines in Idiopathic Heavy Menstrual Bleeding: A Systematic Review. Phytother. Res..

[11] Haznedaroglu B.Z., Haznedaroglu I.C., Walker S.L., Bilgili H., Goker H., Kosar A., Aktas A., Captug O., Kurt M., Özdemir O. (2010). Ultrastructural and Morphological Analyses of the In Vitro and In Vivo Hemostatic Effects of Ankaferd Blood Stopper. Clin. Appl. Thromb. Hemostasis.

[12] Marigold (*Calendula officinalis* L.). https://www.tandfonline.com/doi/abs/10.1080/J157v06n03_08.

[13] Pal K., Kundu S.K., Mandal M.K., Kundu T.K. (2015). Prothrombin time test, Phytochemical screening, Thin layer chromatographic study and Quantitative study of Calendula officinalis leaves. Int. J. Pharmacol. Pharm. Sci..

[14] Scherer W., Gultz J., Lee S.S., Kaim J. (1998). The ability of an herbal mouthrinse to reduce gingival bleeding. J. Clin. Dent..

[15] Chandran P.K., Kuttan R. (2008). Effect of Calendula officinalis Flower Extract on Acute Phase Proteins, Antioxidant Defense Mechanism and Granuloma Formation During Thermal Burns. J. Clin. Biochem. Nutr..

[16] Zitterl-Eglseer K., Sosa S., Jurenitsch J., Schubert-Zsilavecz M., Della Loggia R., Tubaro A., Bertoldi M., Franz C. (1997). Anti-oedematous activities of the main triterpendiol esters of marigold (*calendula officinalis*, L.). J. Ethnopharmacol..

[17] Parente L.M., Andrade M.A., Brito L.A., Moura V.M., Miguel M.P., Lino-Júnior R.D.S., Tresvenzol L.F., Paula J.R., Paulo N.M. (2011). Angiogenic activity of *calendula officinalis* flowers, L. in rats. Acta Cir. Bras..

[18] Ukiya M., Akihisa T., Yasukawa K., Tokuda H., Suzuki T., Kimura Y. (2006). Anti-inflammatory, anti-tumor-promoting, and cytotoxic activities of constituents of marigold (*calendula officinalis*) flowers. J. Nat. Prod..

[19] Halkai K.R., Mudda J.A., Shivanna V., Patil V., Rathod V., Halkai R. (2019). Cytotoxicity evaluation of fungal-derived silver nanoparticles on human gingival fibroblast cell line: An in vitro study. J. Conserv. Dent. JCD.

[20] Auddy B., Ferreira M., Blasina F., Lafon L., Arredondo F., Dajas F., Tripathi P.C., Seal T., Mukherjee B. (2003). Screening of antioxidant activity of three Indian medicinal plants, traditionally used for the management of neurodegenerative diseases. J. Ethnopharmacol..

[21] Tamer T.M., Sabet M.M., Omer A.M., Abbas E., Eid A.I., Mohy-Eldin M.S., Hassan M.A. (2021). Hemostatic and antibacterial PVA/Kaolin composite sponges loaded with penicillin–streptomycin for wound dressing applications. Sci. Rep..

[22] Menda J.P., Reddy T., Deepika R., Sastry T.P. (2014). Preparation and characterization of wound healing composites of chitosan, aloe vera and calendula officinalis—A comparative study. Am. J. Phytomedicine Clin. Ther..

[23] Luo Y., Kirker K.R., Prestwich G.D. (2000). Cross-linked hyaluronic acid hydrogel films: New biomaterials for drug delivery. J. Control. Release.

[24] Dos Santos T.C., Hernández R., Rescignano N., Boff L., Reginatto F.H., Simões C.M., de Campos A.M., Mijangos C. (2018). Nanocomposite chitosan hydrogels based on PLGA nanoparticles as potential biomedical materials. Eur. Polym. J..

[25] El-Hashemy M.A., Sallam A. (2020). The inhibitive action of Calendula officinalis flower heads extract for mild steel corrosion in 1 M HCl solution. J. Mater. Res. Technol..

[26] Arana L., Salado C., Vega S., Aizpurua-Olaizola O., de la Arada I., Suarez T., Usobiaga A., Arrondo J.L., Alonso A., Goñi F.M. (2015). Solid lipid nanoparticles for delivery of Calendula officinalis extract. Colloids Surf. B Biointerfaces.

[27] Wang Q., Chen J., Wang D., Shen M., Ou H., Zhao J., Chen M., Yan G., Chen J. (2021). Rapid Hemostatic Biomaterial from a Natural Bath Sponge Skeleton. Mar. Drugs.

[28] Hossein N., Abolfazl M., Mahdi S., Ali K. (2013). Effect of Cinnamon zeylanicum essence and distillate on the clotting time. J. Med. Plants Res..

[29] Kee N.L., Mnonopi N., Davids H., Naude R.J., Frost C.L. (2008). Antithrombotic/anticoagulant and anticancer activities of selected medicinal plants. Afr. J. Biotechnol..

[30] Hess J.R., Brohi K., Dutton R.P., Hauser C.J., Holcomb J.B., Kluger Y., Mackway-Jones K., Parr M., Rizoli S.B., Yukioka T. (2008). The coagulopathy of trauma: A review of mechanisms. J. Trauma Acute Care Surg..

[31] Rubin R., Strayer D.S., Rubin E. (2011). Rubin’s Pathology: Clinicopathologic Foundations of Medicine.

[32] Preethi K.C., Kuttan G., Kuttan R. (2009). Anti-inflammatory activity of flower extract of Calendula officinalis Linn. and its possible mechanism of action. Indian J. Exp. Biol..

[33] Lagarto A., Bueno V., Guerra I., Valdés O., Vega Y., Torres L. (2011). Acute and subchronic oral toxicities of Calendula officinalis extract in Wistar rats. Exp. Toxicol. Pathol..

[34] Shafeie N., Naini A.T., Jahromi H.K. (2015). Comparison of Different Concentrations of Calendula Officinalis Gel on Cutaneous Wound Healing. Biomed. Pharmacol. J..

[35] Saini P., Al-Shibani N., Sun J., Zhang W., Song F., Gregson K.S., Windsor L.J. (2012). Effects of Calendula officinalis on human gingival fibroblasts. Homeopathy.

[36] Matysik M.G., Wójciak-Kosior M., Paduch R. (2005). The influence of Calendulae officinalis flos extracts on cell cultures, and the chromatographic analysis of extracts. J. Pharm. Biomed. Anal..

[37] Rodríguez-Acosta H., Tapia-Rivera J.M., Guerrero-Guzmán A., Hernández-Elizarraráz E., Hernández-Díaz J.A., Garza-García J.J.O., Pérez-Ramírez P.E., Velasco-Ramírez S.F., Ramírez-Anguiano A.C., Velázquez-Juárez G. (2022). Chronic wound healing by controlled release of chitosan hydrogels loaded with silver nanoparticles and calendula extract. J. Tissue Viability.

[38] El-Sayed M.K., Hommos A.M., Kotry G.S., Labib G.S. (2021). The effect of a calendula based topical formula versus oxidized regenerated cellulose on palatal wound healing: A randomized controlled clinical trial. Alex. Dent. J..

[39] de Brito Sousa J.D., Sachett J.A.G., de Oliveira S.S., Mendonça-da-Silva I., Marques H.O., de Lacerda M.V.G., Fan H.W., Monteiro W.M. (2018). Accuracy of the Lee-White Clotting Time Performed in the Hospital Routine to Detect Coagulopathy in Bothrops atrox Envenomation. Am. J. Trop. Med. Hyg..

[40] Ogle C.W., Dai S., Ma J.C. (1976). The haemostatic effects of the Chinese herbal drug Yunnan Bai Yao: A pilot study. Am. J. Chin. Med..

[41] Ebrahimi F., Torbati M., Mahmoudi J., Valizadeh H. (2020). Medicinal Plants as Potential Hemostatic Agents. J. Pharm. Pharm. Sci..

[42] Kooshki F., Tabatabaei F.S., Tajik S., Aayan A. (2018). The comparison of antimicrobial effects of herbal and chemical agents on toothpaste: An experimental study. Dent. Res. J..

[43] Scarborough J.E., Schumacher J., Pappas T.N., McCoy C.C., Englum B.R., Agarwal S.K.J., Greenberg C.C. (2016). Which Complications Matter Most? Prioritizing Quality Improvement in Emergency General Surgery. J. Am. Coll. Surg..

[44] Asti A., Gioglio L. (2014). Natural and synthetic biodegradable polymers: Different scaffolds for cell expansion and tissue formation. Int. J. Artif. Organs..

[45] Mp S.K. (2016). Local hemostatic agents in the management of bleeding in oral surgery. Asian J. Pharm. Clin. Res..

[46] AshwlayanVD K.A., Verma M. (2018). Therapeutic potential of Calendula officinalis. Pharm. Pharmacol. Int. J..

[47] Della Loggia R., Tubaro A., Sosa S., Becker H., Saar S., Isaac O. (1994). The role of triterpenoids in the topical anti-inflammatory activity of Calendula officinalis flowers. Planta Med..

[48] Khoshmohabat H., Paydar S., Kazemi H.M., Dalfardi B. (2016). Overview of Agents Used for Emergency Hemostasis. Trauma Mon..

